# Association of Ca×PO4 product with levels of serum C-reactive protein in regular hemodialysis patients

**DOI:** 10.12861/jrip.2012.20

**Published:** 2012-09-01

**Authors:** Hamid Nasri

**Affiliations:** Department of Nephrology, Shahrekord University of Medical Sciences, Shahrekord, Iran

**Keywords:** Hyperphosphatemia, End-stage renal disease, Hemodialysis

## Abstract

**Introduction:** Numerous studies have attempted to identify risk factors for mortality and morbidity in maintenance hemodialysis patients. In this study we sought to examine the association of the levels of serum C-reactive protein (CRP) with value of Ca×PO4 product, in stable hemodialysis patients.

**Patients and Methods:** Based on the severity of secondary hyperparathyroidism, patients being treated with oral active vitamin D3, calcium carbonate/Renagel tablets at various doses. Fasting serum 25-hydroxy vitamin D and intact serum parathormone and also serum blood urea nitrogen, CRP, calcium, phosphorus, alkaline phosphatase was measured.

** Results:** A total of 41 patients, enrolled to the study. The mean patients’ age were 46(17.6) years. The value of serum CRP of patients was 8.6 (6.6) mg/l (median 6 mg/l). The value of Ca×PO4 product was 50.5(15.5) mg2/dl2 (median: 50 mg^2^/dl^2^). In this study, a significant inverse association between Ca×PO4 product and the age of the patients was seen. A significant positive correlation of logarithm of serum CRP with Ca×PO4 product was found.

**Conclusion:** The result of this study, revealed the need to further attention to hyperphosphatemia in hemodialysis patients.

**Implication for health policy/practice/research/medical education:**

In a study on 41stable hemodialysis patients, we found a significant positive correlation of serum C-reactive protein with Ca×PO4 product. The result of this study, revealed the need to further attention to hyperphosphatemia in hemodialysis patients.

## Introduction

Various investigations have attempted to identify risk factors for mortality and morbidity in hemodialysis patients. Most have shown important relations among demographic factors such as older age, male gender, white race and mortality (1,2). Comorbid situations like diabetes, cardiovascular disease and nutritional status (e.g., serum albumin, creatinine) have also been consistently associated with mortality and morbidity (3-5). In fact one of the factors which potentially connect to the mortality and morbidity is the control of mineral metabolism (6). The mechanisms underlying the increase in mortality and morbidity associated with disturbances in mineral metabolism remain speculative. Several reports have described the ability of serum phosphorus to potently stimulate the phenotypic transformation of vascular smooth muscle cells into osteoblasts capable of producing a pro-mineralizing milieu (7,8). In this state, supersaturatinion of extracellular calcium and phosphorus may promote the development of medial wall vascular calcification, a pathologic process recognized to be associated with increases in arterial stiffness, aortic pulse wave velocity, left ventricular size, and all-cause mortality in patients on hemodialysis (9). Elevated serum phosphorus level is a predictable adjunct of end-stage kidney failure in the absence of dietary phosphate restriction or supplemental phosphate binders. The results of hyperphosphatemia comprise the development and progression of secondary hyperparathyroidism and a predisposition to metastatic calcification when the product of serum calcium and phosphorus (Ca×PO4) is raised. Both of these circumstances may contribute to the substantial morbidity and mortality found in patients with end-stage kidney failure (6). Recently, a strong emphasis has been directed on elevated C-reactive protein (CRP) levels as an important risk factor for morbidity and mortality too (10-12). Furthermore, an association has been noted between elevated CRP levels, atherosclerotic plaques and malnutrition (12). Ectopic calcifications are defined as a process of inappropriate biomineralization occurring in soft tissues. They are typically composed of calcium salts. In hemodialysis patients such a condition referred to as metastatic calcification, which is said to be responsible for progressive cardiac and vascular injury, leading to invalidating clinical complications and increased mortality risk (13). Considering that the amount of coronary calcium is a marker of atherosclerosis (14), while atherosclerosis is at present viewed as an inflammatory disease (15) and while, CRP is a marker of cardiovascular risk both in non-uremic (16) and uremic subjects (17) that hyperphosphatemia and elevated calcium–phosphate product are associated with an increased risk of death (6).

## Objectives

This study was aimed to elucidate whether and how in end-stage kidney failure patients on hemodialysis the levels of CRP correlate with Ca×PO4 product.

## Patients and Methods

### Patients

This cross-sectional study was conducted on patients with end-stage kidney failure patients who were undergoing regular hemodialysis. According to the intensity of secondary hyperparathyroidism, each patient being treated for secondary hyperparathyroidism was given oral active vitamin D3 (Calcitriol; Rocaltrol) (Roche Hexagon; Roche Laboratories Inc, New Jersey, USA ), calcium carbonate tablets and Renagel (sevelamer; Genzyme Europe B.V.; United Kingdom/Ireland) tablet at various doses. According to the severity of anemia, patients were prescribed intravenous iron therapy with iron Sucrose (Venofer; Vifor (international) Inc. St. Gallen/Switzerland) at various doses after each dialysis session. All patients received treatments of 5 mg folic acid daily, oral vitamin B-complex tablets daily, and 2,000 U intravenous Eprex (recombinant human erythropoietin [Rhuepo] (Janssen-Cilag; CILAG-AG International 6300 Zug/Switzerland) after each dialysis session. Exclusion criteria were active or chronic infection and using drugs had adverse effects on bone marrow.

### Laboratory tests

Complete blood counts were measured using Sysmex-KX-21N cell counter (SYSMEX CORPORATION; Mundelein, Illinois, Sysmex America, Inc.). Serum 25-hydroxy (25-OH) Vitamin D level (normal range of values is 25 to 125 nmol/L) and intact serum PTH (iPTH) were measured as follows. Blood samples were obtained after an overnight fast, blood sample were centrifuged within 15 min of venipuncture, and were measured by enzyme-linked immunosorbent assay (ELISA) method using DRG kits of Germany. Intact serum PTH (iPTH) was measured by the radioimmunoassay (RIA) method using DSL-8000 kits of USA (normal range of values is 10-65 pg/ml). Also peripheral venous blood samples were collected after an overnight fast, for biochemical analysis including serum post and predialysis blood urea nitrogen (BUN), serum C-reactive protein (CRP), albumin (Alb), serum calcium (Ca), phosphorus (P) and alkaline phosphatase (ALP) and also serum ferritin (by radio immune assay method; RIA) were measured using standard methods. Body mass index (BMI) calculated using the standard formula (post-dialyzed weight in kilograms/height in square meters; kg/m^2^). Duration and dosages of hemodialysis treatment were calculated from the patients’ records. The duration of each hemodialysis session was 4 hours. For statistical analysis, the data are expressed as the mean ± standard deviation (SD) and median values. Comparison between the groups was done using Student’s *t*-test. Statistical correlations were assessed using partial correlation test. CRP was Ln-transformed in all statistical analyses because of positive skewness.

### Ethical issues

1) The research followed the tenets of the Declaration of Helsinki; 2) informed consent was obtained; 3) the research was approved by the institutional review board.

### Statistical analysis

All statistical analyses were performed using SPSS 11.5 (SPSS Inc., Chicago, IL). Statistical significance was determined at a P value <0.05.

## Results

There was a total of 41 patients (15 women, 26 men), consisting of 29 non-diabetic hemodialysis patients and 12 diabetic hemodialysis patients. Table 1 illustrated the patients’ mean (SD) age, the length of time they were on hemodialysis, the dialysis dosage, and the results of laboratory tests. The mean patient age was 46 (17.6) years. The value of serum CRP of patients was s8.6 mg/l (6.6) (median 6 mg/l). The value of Ca×PO4 product was 50.5 (15.5) mg^2^/dl^2^ (median: 50 mg^2^/dl^2^). In this study no significant difference of Ca×PO4 product between male or females and diabetics or non-diabetics was seen (P >0.05). While a significant difference of serum CRP between diabetic and non-diabetics was found (P= 0.014), no significant difference of serum CRP between male or female groups was seen (P >0.05) .No significant relationship between Ca×PO4 product and duration and doses of dialysis was found In this study a significant inverse correlation between Ca×PO4 product and the age of the patients (r= -0.34, P= 0.034) (adjusted for duration and doses of dialysis) was detected. Also, a significant positive correlation between logarithm of serum CRP with age (r= 0.42, P= 0.011) (adjusted for duration and doses of dialysis and serum P, iPTH and also ferritin) was found. A significant positive correlation of logarithm of serum CRP with Ca×PO4 product(r= -0.33, P= 0.047) (adjusted for age, duration and doses of dialysis and) was found.

**Table 1:** Data of the patients.

**Total patients** **n=41**	**Minimum**	**Maximum**	**Mean±SD**	**Median**
**Age ( years)**	16	80	46 ±17.6	46
DH* (months)	2	156	29.5±35	29.5
**Dialysis amount (Sessions)**	18	1584	269±375	153
**URR (%)**	39	76	58.7±8.75	58
**CRP (mg/l)**	3	40	8.6±6.6	6
**Hgb (g/dl)**	5	13	9±2	9
**25-hydroxy D (nmol/l)**	0.3	36	7.6±9.5	3.2
**iPTH (** *P* **g/ml)**	16	1980	408±440	250
**Alb (g/dl)**	2.4	5	3.85±0.53	4
**Ferritin ( ng/dl)**	35	1250	497±287	420
**Ca(mg/dl)**	5	10	7.7±0.99	8
**P(mg/dl)**	3	10	6.3±1.9	6.4
**Alp (IU/l)**	150	5487	632±878	428
**Ca** **×** **PO4 (mg** ^2^ **/dl** ^2^ ** )**	21	80	50.5±15.5	50
**BMI (kg/m** ^2^ **)**	16	34	21.4±4.3	20
*Duration of hemodialysis				

## Discussion

In the present study we found a significant inverse correlation between Ca**×**PO4 product and the ages of the patients and a significant positive correlation between of serum CRP with age. Furthermore significant positive correlation of serum CRP with Ca**×**PO4 product was found too. In a study conducted by Block *et al*. found thatserum phosphate levels >6.5 mg/dl and a calcium–phosphateion product >72 mg^2^/dl^2^ are associated with an 18–39%higher risk of death, compared with normal reference groups(namely a serum phosphate of 4.4–5.5 mg/dl and calcium–phosphateproduct of 43–52 mg^2^/dl^2^) (6). Block *et al*. fordetermining associations among disorders of mineral metabolism,mortality, and morbidity in hemodialysis patients, data on 40,538hemodialysis patients with at least one determination of serumphosphorus and calcium during the last 3 mo of 1997 were analyzed. Whenexamined collectively, the population attributable risk percentagefor disorders of mineral metabolism was 17.5%, owing largelyto the high prevalence of hyperphosphatemia. Hyperphosphatemiaand hyperparathyroidism were significantly associated with all-cause,cardiovascular, and fracture-related hospitalization. They concluded that disordersof mineral metabolism are independently correlated with mortalityand morbidity associated with cardiovascular disease and fracturein patients on hemodialysis (18). Marco *et al*. studied on one hundred and forty-three hemodialysis patients which were followed for six years. They showed an increased risk of cardiovascular death in patients with serum P >6.5 mg/dl, PTH >50 *p*mol/l , Ca**×**P>52 (19). High levels of CRP , in renal failure patients , have been shown, and high CRP levels in dialysis patients are predictive of futurecardiovascular events (20-25). Inflammation might bea trigger for calcium deposition in the arteries of dialysispatients, peculiarly at the end of each dialysis session whenback-filtration might occur, plasma calcium concentrations reachtheir maximum levels and the patients become alkalotic*.*In this situation it should also be considered that CRP, beinga member of the pentraxin family, binds to damaged tissue ina calcium-dependent manner and shows membrane association withmultiple calcium ions (26) Moreover, CRP binds to enzymaticallydegraded LDL particles in early atherosclerotic lesions,inducing complement activation and promoting the developmentand progression of the atherosclerotic lesion (27).The increase of CRP in stable dialysis patientsmay be due to the stimulation of monocytes/macrophages by dialysatecontaminants and, in turn, may promote by itself atheroscleroticchanges in the cardiovascular tree. Concomitant to such an inflammatorystate other cofactors are at work: a high oral intake of calciumsalts and an excessive vitamin D therapy in a daily fashionand a positive calcium balance due to supra-normal calcium inthe dialysate in an intermittent fashion. In this case metabolicalkalosis may have an additional role in calcium precipitation (26,27). Our study detect an inverse association of dialysis adequacy with both serum CRP and Ca**×**PO4 product, indicates that an adequate hemodialysis on one hand will be associated with lower Ca**×**PO4 product and on the other hand diminishes the inflammatory state of uremia (28,29). We could find a positive correlation between Ca**×**PO4 product and serum CRP level. To detect the clinical importance of this association, Movilli *et al*. conducted a study on 47 uremic patients (age 65) on regular hemodialysis. Their patients had no clinical evidence of either acute infectious or inflammatory diseases for at least 4 weeks before the study. They were on regular bicarbonate hemodialysis for 6-329 months (median 42). A significant hyperbolic correlation between Ca**×**PO4 and CRP was observed. A piecewise linear regression model analysis identified a break-point for Ca**×**PO4 at 55 mg^2^/dl^2^. They showed that in chronic hemodialysis patients in steady clinical conditions with no clinical evidence of either infectious or inflammatory diseases, a high Ca**×**PO4 is associated with high CRP concentrations and intensive lowering of Ca**×**PO4, reduces CRP (30). In this view, inflammation might bea trigger for calcium deposition in the arteries of dialysispatients, peculiarly at the end of each dialysis session whenback-filtration might occur, plasma calcium concentrations reachtheir maximum levels and the patients become alkalotic (28-30). Therefore an elevated Ca**×**PO4 product and C-reactive protein have been associated with coronary artery calcification and increased cardiovascular mortality in hemodialysis patients. In fact, the view of the clinical problems associated with secondaryrenal hyperparathyroidism in end-stage kidney failurepatients has changed considerably in the last few years.While it formerly was considered primarily a skeletal disorder,recent data show strong associations between renal secondary hyperparathyroidism andboth disturbed bone/mineral metabolism and the development ofcardiovascular calcifications, leading to cardiovascular morbidityand patient mortality (6,18,31-33). Our findings showed the need to further attention to hyperphosphatemia and uncontrolled secondary hyperparathyroidism in maintenance hemodialysis patients.

## Author’s contribution

HN is the single author of the manuscript.

## Conflict of interests

The author declared no competing interests.

## Ethical considerations

Ethical issues (including plagiarism, data fabrication, double publication) have been completely observed by the author.

## Funding/Support

None.
